# Characterisation of RT Connection and RNase H Polymorphisms in HIV-1 Subtype C in Botswana

**DOI:** 10.3390/v18040434

**Published:** 2026-04-03

**Authors:** Boitumelo J. L. Zuze, Wonderful T. Choga, Natasha O. Moraka-Mankge, Ontlametse T. Choga, Lynnette Bhebhe, Dorcas Maruapula, Thato Phuthego, Margaret Mokomane, Sikhulile Moyo, Simani Gaseitsiwe

**Affiliations:** 1 Botswana Harvard Health Partnership, Private Bag BO320, Bontleng, Gaborone, Botswana; bzuze@bhp.org.bw (B.J.L.Z.); wchoga@bhp.org.bw (W.T.C.); nmoraka@bhp.org.bw (N.O.M.-M.); otbareng@bhp.org.bw (O.T.C.); lynnettebhebhe@gmail.com (L.B.); dmaruapula@bhp.org.bw (D.M.); smoyo@bhp.org.bw (S.M.); 2 Department of Medical Laboratory Sciences, School of Allied Health Sciences, University of Botswana, Gaborone, Botswana; info@ub.ac.bw (T.P.); mokomanem@ub.ac.bw (M.M.); 3 Department of Immunology and Infectious Diseases, Harvard T. H. Chan School of Public Health, Boston, MA 02115, USA; 4 Department Pathology, Division of Medical Virology, Faculty of Medicine and Health Sciences, Stellenbosch University, Tygerberg, Cape Town 7505, South Africa; 5 School of Health Systems and Public Health, University of Pretoria, Pretoria 0002, South Africa

**Keywords:** HIV-1C, reverse transcriptase inhibitors (RTIs), reverse transcriptase connection (RT-conn), ribonuclease H (RNase H), drug resistance mutations (DRMs), polymorphisms, Botswana

## Abstract

Emerging evidence suggests that polymorphisms in the reverse transcriptase connection (RT-conn) and RNase H domains may contribute to resistance to reverse transcriptase inhibitors (RTIs). Here, we characterised the polymorphic landscape of the RT-conn and RNase H domains in HIV-1 subtype C (HIV-1C) from Botswana across the pre-ART and post-ART eras, including treatment-naïve (TN) and treatment-experienced (TE) individuals. A total of 1571 HIV-1C sequences were analysed: 76 pre-ART (≤2002) and 1495 post-ART (>2002) sequences were obtained from the Los Alamos database and the Botswana Combination Prevention Project (2013–2018). Post-ART sequences were stratified into TN (n = 1282) and TE individuals with virologic failure (TEVF, n = 213). Naturally occurring and ART-associated polymorphisms within RT-conn (aa 321–440) and RNase H (aa 441–560) were assessed. Among TN individuals, 12 polymorphisms exceeded 5% pre-ART, including R461K and L491P, while 31 polymorphisms were observed post-ART, indicating a temporal shift. Several substitutions were significantly higher in TEVF and showed a history of thymidine analogue-, tenofovir- and lamivudine/emtricitabine-based exposure. Covariant analysis identified significant co-occurrence of polymerase mutations (M184V/I, D67N) with RT-conn/RNase H substitutions (*p* < 0.05). These findings demonstrate HIV-1C evolution within the extended RT domains under ART pressure and support their inclusion in molecular surveillance frameworks in Botswana.

## 1. Introduction

The introduction and expansion of antiretroviral therapy (ART) has led to significant reduction in human immunodeficiency virus (HIV)-related morbidity and mortality globally [1,2]. However, the long-term effectiveness of ART is increasingly threatened by the emergence and transmission of HIV drug resistance, especially in low- and middle-income countries (LMICs), where large numbers of people with HIV (PWH) are in need of lifelong treatment [3]. Most of the HIV drug resistance research has focused on HIV-1 subtype B (HIV-1B), which is predominantly found in high-income settings [4,5,6,7,8]; this is despite the global dominance of HIV-1 subtype C (HIV-1C), which still remains under-characterised. As subtype-specific differences in resistance pathways have been noted [9], there has been an increase in research to better understand the resistance mechanisms of non-B subtypes.

HIV reverse transcriptase (RT) plays a key role in viral replication and is targeted by three classes of licenced antiretroviral drugs: nucleoside RT inhibitors (NRTIs), non-nucleoside RT inhibitors (NNRTIs), and nucleoside RT translocation inhibitors (NRTTIs). The RT structure consists of three domains: the polymerase, the connection (RT-conn), and the ribonuclease H (RNase H). While most well-characterised HIV drug resistance mutations (DRMs) are found in the polymerase domain due to direct drug targeting [10,11], there is evidence that residue changes in the RT-conn and RNase H domains may contribute to resistance through indirect mechanisms [12,13,14]. Substitutions in these regions have been shown to influence the balance between polymerase and RNase H activities, potentially reducing template degradation and allowing additional time for nucleotide excision or continued DNA synthesis, which may enhance resistance to nucleoside analogues or alter polymerase processivity [12,13,14], thus contributing to reducing susceptibility to NRTIs and NNRTIs.

Several studies conducted in HIV-1B have shown that amino acid substitutions in the RT-conn and RNase H domains can contribute to resistance to RT inhibitors [14], with specific substitutions such as D549N, Q475A, and Y501A shown to reduce RNase H cleavage efficiency and increase resistance to certain NNRTIs, including nevirapine (NVP) and delavirdine (DLV). However, these effects are not consistent across all drugs, suggesting intra-subtype variability and potential subtype-dependent differences. Subtype-specific resistance-associated substitutions have also been reported in the RT-conn and RNase H domains, but their variable frequencies across subtypes highlight inconsistent resistance patterns [15,16]. However, studies examining these domains in HIV-1C remain scarce [4,6,17], and their clinical relevance is yet to be fully defined.

To address these gaps, this study investigated the polymorphic landscape of the RT-conn and the RNase H domains in HIV-1C sequences from Botswana. These analyses aimed to: characterise HIV-1C-specific polymorphisms in the RT-conn and RNase H domains; identify polymorphisms potentially associated with drug resistance; and inform more effective ART regimen design, resistance surveillance, and treatment strategies tailored to the HIV-1C epidemic in Botswana.

## 2. Materials and Methods

### 2.1. Study Design and Population

This was a retrospective observational study employing a comparative cross-sectional design to analyse HIV-1C sequences from three distinct groups. No new sequencing was performed for the purpose of this study. All sequences analysed were previously generated as part of the Botswana Combination Prevention Project (BCPP) study or obtained from the Los Alamos HIV Sequence Database (https://www.hiv.lanl.gov/content/sequence/HIV/mainpage.html, accessed on 2 December 2025).

Seventy-six sequences representing baseline natural polymorphisms from the pre-ART era were obtained from the Los Alamos database, comprising samples collected before or during 2002. For the post-ART rollout period, previously generated sequences from 1282 TN individuals and 213 TE individuals with virologic failure (VF) from the BCPP cohort were analysed. Sequences from the BCPP study are available through a controlled data-sharing framework in accordance with the study’s ethical approvals and data access polices.

The objective of this study was to characterise naturally occurring polymorphisms in the RT-conn and RNase H domains and to evaluate potential ART-driven polymorphisms across different treatment exposures and time periods. The analysed post-ART sequences were obtained from a larger dataset of 6078 HIV-1C sequences generated from participants enrolled in the BCPP [11,17,18,19]. From this dataset, sequences of the TN individuals and TE individuals with VF were selected to be included in the present analysis. The BCPP enrolled PWH aged 16–64 years who were randomly selected from 30 communities across northern, central and southern Botswana. The cohort included both HIV-positive and HIV-negative individuals, with HIV status determined through documented evidence (e.g., HIV test results, ART prescriptions) or household-based HIV testing conducted following Botswana’s national guidelines, which utilise two rapid HIV tests performed in parallel.

Most ART-experienced individuals in the BCPP cohort had been initiated on efavirenz (EFV)- or nevirapine (NVP)-based regimens, which represented the standard first-line therapy prior to the nationwide adaptation of dolutegravir (DTG)-based regimens in June 2016. In this study, only TN individuals from both pre- and post-ART rollout periods were initially analysed to assess the natural polymorphic landscape of HIV-1C in Botswana, providing a baseline comparative analysis of polymorphisms in the RT-conn and RNase H domains. Subsequent analyses focused on assessing the impact of ART exposure by comparing TN individuals to TE individuals with VF, thereby assessing the ART-driven polymorphic patterns within the BCPP cohort.

### 2.2. Previously Generated Sequence Data

The near-full-length HIV-1 sequences analysed in this study were generated previously as part of the BCPP. Detailed amplification and sequencing methods have been described elsewhere [20].

### 2.3. Selection of Study Participants

**Scheme 1:**
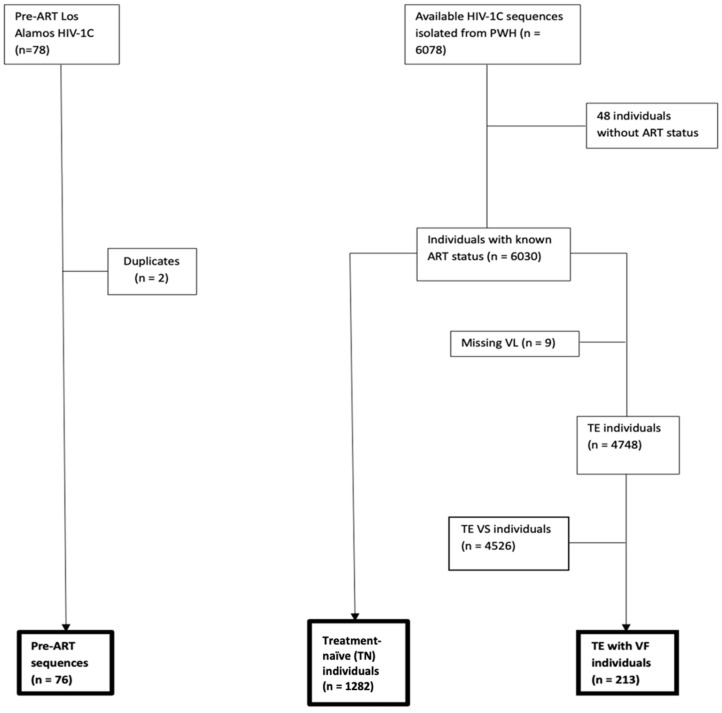
This is a schema used to perform data sorting and shows a proportion of sequences with missing clinical data that were not included in the current analyses. ART—Antiretroviral therapy, n—Population size, PWH—People with HIV, TN—Treatment-naïve, TE—Treatment-experienced, VL—Viral load, VS—Virally suppressed, VF—Virologic failure.

Participants for this study were selected from the BCPP cohort, consisting of PWH who had available HIV-1 sequences covering RT-conn and RNase H domains, along with documented HIV-1 viral load (VL) measurements at their first study visit. VL was quantified using an Abbott m2000sp/rt assay (Wiesbaden, Germany) with a range of 4 × 10^1^–1 × 10^7^ copies/mL [19]. Participants were categorised based on ART status into TN and TE with VF groups. TN individuals had no prior ART exposure at the time of sampling, while TE individuals with VF had documented ART use and VF, which is defined as VL > 400 copies/mL at the time of sample collection. Additionally, HIV sequences from PWH covering RT-conn and RNase H domains obtained prior to Botswana’s national ART rollout were included as well (Scheme 1).

### 2.4. Mutational Analysis

A total of 1573 HIV-1C sequences were available for analysis. These included 78 pre-ART sequences from the Los Alamos database, 1282 TN and 213 TE with VF post-ART sequences. Following control checks, 1571 sequences remained for final analysis as 2 pre-ART sequences were duplicates. We used the Gene cutter tool (https://www.hiv.lanl.gov/content/sequence/GENE_CUTTER/cutter.html, accessed on 2 December 2025) to extract the RT and RNase H fragment of the HIV pol gene. The obtained nucleotides were aligned using NextAlign [21] and HXB2 as HIV reference sequence. The subsequent multiple-sequence alignment (MSA) was visually inspected in Aliview before translating to the corresponding amino acids, which were then used for sequence analysis. After quality control checks, only 1571 sequences qualified for analysis. We developed an in-house Python v3.13 script to curate all mutations within the MSA. The wild-type mutations were based on the HXB2.

To explore the potential associations between identified polymorphisms and antiretroviral exposure, substitutions that were significantly enriched in TE individuals with VF were further evaluated in relation to documented treatment regimens within the BCPP study. Multivariate logistic regression was used to determine the residue prevalence between TN and TE with VF individuals to identify polymorphisms potentially associated with ART-selective pressure. Identified polymorphisms were subsequently interpreted in the context of the predominant NRTI-based regimen used in the study (including zidovudine, tenofovir, lamivudine and emtricitabine).

### 2.5. Statistical Analysis

Baseline demographic and clinical characteristics (available only for post-ART participants) were summarised using descriptive statistics. Categorical variables were reported as percentages, while continuous variables were summarised using medians and interquartile ranges (IQRs). Comparisons between TN and TE individuals with VF were performed using the Wilcoxon rank-sum test for the continuous variables (e.g., VL, age) and the Chi-square test for categorical variables (e.g., district, gender).

The prevalence of DRMs across study groups was estimated with 95% confidence intervals using binomial exact method. Group comparisons of DRM prevalence were performed using the test of proportions.

To identify polymorphisms associated with VF, substitution frequencies at each amino acid position across the RT-conn and RNase H domains were compared between TN individuals and TE individuals with VF using Fisher’s exact test. Logistic regression was used to quantify the enrichment of polymorphisms in TE individuals with VF. To account for multiple testing across amino acid positions, *p*-values were adjusted using the Bonferroni correction. Polymorphic enrichment across the RT region was visualised using Manhattan plots generated in R, where each amino acid position (based on HXB2 numbering) was plotted against the −log_10_ (*p*-value) obtained from the association tests. Polymorphisms exceeding the Bonferroni-corrected significance threshold were considered significantly associated with VF.

Covariation analysis was performed to investigate potential interactions between mutations across the RT region. Known RT polymerase drug resistance mutations (M184V, M184I and D67N) were used as anchor mutations to identify substitutions that significantly co-occurred within the RT-conn and RNase H domains. Pairwise associations between anchor mutations and other substitutions were evaluated using Fisher’s exact test and mutation pairs with statistically significant co-occurrence were considered covariant. Significant covariant pairs were subsequently visualised using network analysis, where nodes represented individual amino acid substitutions and edges represented statistically significant covariation relationships.

All statistical analyses were conducted using STATA version 15 software (Stata Corp LP, College Station, TX, USA) and Rstudio Version 2025.05.0 + 496 (Posit Software, PBC), with a *p*-value ≤ 0.05 considered to be statistically significant.

## 3. Results

### 3.1. Participant Characteristics

A total of 1495 HIV-1 near-full-length sequences were analysed from the BCPP study. Of the 1495 participants, 1282 participants were TN and 213 were TE with VF (detectable VL > 400 copies/mL at entry). The median age at enrolment was 34 years (Q1, Q3: 27.0, 42.0), 70.8% were female and the median log_10_ viral load was 4.23 (Q1, Q3: 3.45, 4.80); these characteristics had no significant difference in the TN and TE with VF groups. However, the district category showed statistical significance amongst the three districts (northern, southern and central), with the central district having the highest number of individuals included in the analysis (Table 1).

### 3.2. Naturally Occurring Polymorphism Variation in HIV-1C in Botswana Before ART Rollout

**Figure 1:**
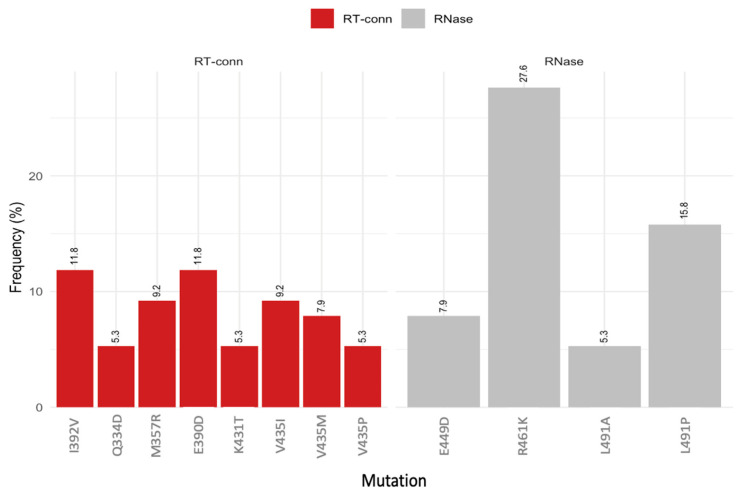
Baseline frequencies of polymorphisms (>5%) in RT-conn and RNase H domains prior to ART introduction: There were 90 detected amino acid substitutions; 61 were found pre-ART rollout but only 12 had frequencies above 5%. There were 8 from the RT-conn domain (aa 321–440) and 4 from the RNase H domain (aa 441–560). These were R461K, L491P, 1393V, E396D, V435I, M357R, V435M, E449D, V435P, L491A, Q334D and K451T, with R461K having the highest frequency at 27.6%. RT-conn—Reverse transcriptase connection, RNase H—Ribonuclease H.

Figure 1 shows that a total of 12 polymorphisms with frequencies >5% were observed in treatment-naïve individuals sampled prior to ART rollout. The most prevalent polymorphisms were R461K (27.6%) and L491P (15.8%) in the RNase H domain, and I329V (11.8%) and E396D (11.8%) in the RT-conn domain. Additional frequently detected polymorphisms in the RT-conn domain included V435I (9.2%), M357R (9.2%), V435M (7.9%), V435P (5.3%), Q334D (5.3%), and K431T (5.3%). Other RNase H-domain polymorphisms above the threshold included E449D (7.9%) and L491A (5.3%).

### 3.3. Naturally Occurring Polymorphisms Circulating Among Treatment-Naïve Individuals Post-ART Rollout

**Figure 2:**
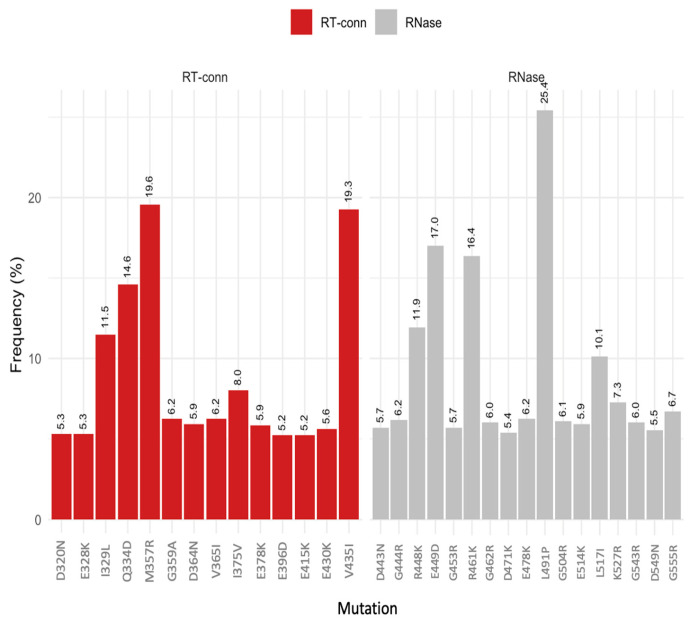
Post-ART frequencies of polymorphisms (>5%) in treatment-naïve individuals: There were 90 detected amino acid substitutions; 60 were found in treatment-naïve individuals post-ART rollout but only 31 had frequencies above 5%. There were 14 from the RT-conn domain (aa 321–440) and 17 from the RNase H domain (aa 441–560). These were L491P, M357R, V435I, E449D, R461K, Q334D, R448K, I329L, L517I, I375V, K527R, G555R, E478K, V365I, G359A, G444R, G504R, G462R, G543R, D364N, E514K, E378K, G453R, D443N, E430K, D549N, D471K, D320N, E328K, E396D and E415K, with L491P having the highest frequency at 25.4%. RT-conn—Reverse transcriptase connection, RNase H—Ribonuclease H.

Among treatment-naïve individuals sampled after ART introduction, 31 polymorphisms were detected at frequencies >5%, reflecting a broader polymorphic spectrum compared to the pre-ART era, as shown in Figure 2. The most prevalent polymorphisms were L491P (25.4%), M357R (19.6%), V435I (19.3%), and E449D (17.0%), spanning both RNase H and RT connection domains. Other notable polymorphisms included R461K (16.4%), Q334D (14.6%), R448K (11.9%) and I329L (11.5%). Several additional polymorphisms appeared at moderate frequencies (5–10%), such as L517I, I375V, K527R and others.

### 3.4. Temporal Evolution of Naturally Occurring Polymorphism in HIV-1C in Botswana in the Pre- and Post-ART Era

**Figure 3:**
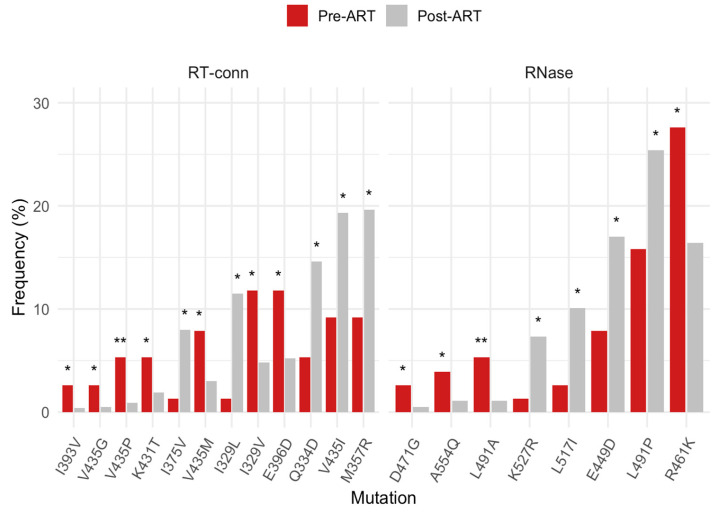
Polymorphisms present in both pre- and post-ART eras in treatment-naïve individuals: There were 90 detected amino acid substitutions; 20 were found in both pre-ART and in post-ART treatment-naïve individuals. There were 12 from the RT-conn domain (aa 321–440) and 8 from the RNase H domain (aa 441–560). These were V435P, L491A, 1393V, I329V, I329L, R461K, V435G, V435M, Q334D, E396D, V435I, M357R, A554Q, I375V, L517I, E449D, D471G, K431T, K527R and L491P. All were statistically significant between these groups (*p*-value < 0.05%) and L491P *p*-value = 0.05%. * indicates statistically significant differences between pre-ART and post-ART sequences (*p* < 0.05). ** indicates statistically significant differences between pre-ART and post-ART sequences (*p* < 0.01) ART—Antiretroviral therapy, RT-conn—Reverse transcriptase connection, RNase H—Ribonuclease H.

**Figure 4:**
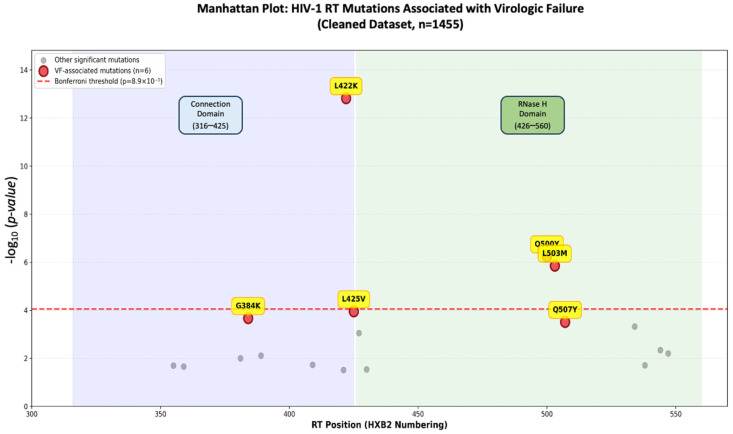
Manhattan plot of RT connection and RNase H polymorphisms associated with virologic failure: Manhattan plot showing the RT connection (aa 316–425) and RNase H (aa 426–560) polymorphisms significantly associated with virologic failure. The *x*-axis represents the RT position (HXB2 numbering) and the *y*-axis shows −log_10_ (*p*-values) derived from Fisher’s exact tests comparing treatment-naïve and treatment-experienced individuals with virologic failure. The dashed horizontal line indicates the Bonferroni-corrected significance threshold. Polymorphisms exceeding this threshold are highlighted and labelled.

A total of 20 polymorphisms were detected in both pre-ART and post-ART treatment-naïve sequences in Figure 3, with frequencies ranging from 0.4% to 27.6% (Figure 4). Two amino acid substitutions, V435P and L491A, were significantly more prevalent in pre-ART sequences (5.3% vs. 0.9% and 5.3% vs. 1.1%, respectively; *p* < 0.01). Several other residues were statistically higher pre-ART (*p* < 0.05), including I393V (2.6% vs. 0.4%), I329V (11.8% vs. 4.8%), R461K (27.6% vs. 16.4%), V435G (2.6% vs. 0.5%), V435M (7.9% vs. 3.0%), E396D (11.8% vs. 5.2%), A554Q (3.9% vs. 1.1%), D471G (2.6% vs. 0.5%) and K431T (5.3% vs. 1.9%). In contrast, I329L, Q334D, V435I, M357R, I375V, L517I, E449D and K527R were significantly higher post-ART (*p* < 0.05), with frequencies ranging from 7.3% to 25.4%. Notably, L491P showed a borderline significant increase post-ART (15.8% vs. 25.4%, *p* = 0.05).

### 3.5. RT Connection and RNase H Polymorphisms Associated with Virologic Failure

To identify RT connection and RNase H polymorphisms significantly associated with VF, a Manhattan plot using a cleaned dataset of TN and TE with VF individuals (n = 1455) was generated to visualise the statistical association between amino acid positions and VF across the RT connection and RNase H domains (Figure 4). Several polymorphisms exceeded the Bonferroni-corrected significance threshold, indicating a strong statistical association with VF. Within the RT connection domain, L422K, G384K, and L425V were among the most significant associations. In the RNase H domain, Q500Y, L503M, and Q507Y demonstrated significant enrichment in TE individuals with VF. These polymorphisms were located within well-defined functional subdomains and showed consistent signals across statistical analyses. Overall, the Manhattan plot highlights discrete loci within the connection and RNase H domains that are significantly associated with VF under ART exposure, supporting the presence of non-random polymorphism patterns associated with VF.

### 3.6. Differential Frequency of RT Connection and RNase H Polymorphisms in Treatment-Experienced Individuals with Virologic Failure

**Figure 5:**
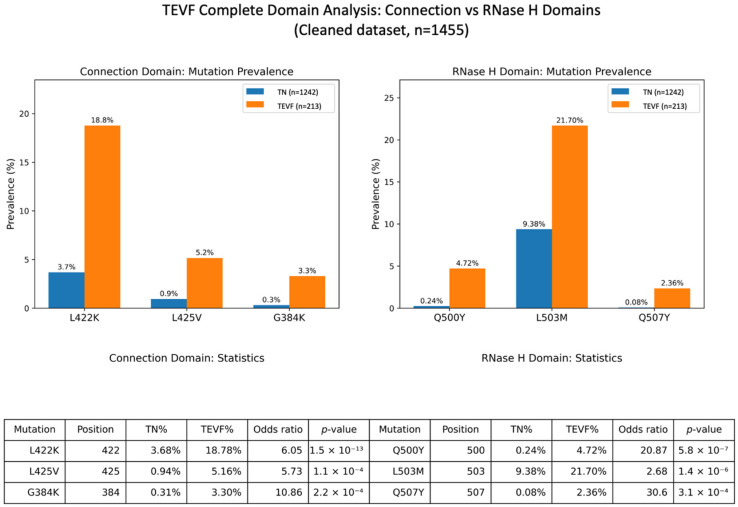
Enrichment of RT connection and RNase H polymorphisms in treatment-experience individuals with virologic failure: There were 49 detected amino acid substitutions found in both post-ART treatment-naïve and treatment-experienced individuals with virologic failure. Only six selected polymorphisms (L422K, L425V and G384K in the RT connection and Q500Y, L503M and Q507Y in the RNase H) identified in the Manhattan plot are shown. Summary tables report polymorphism position, frequency, odds ratios and *p*-values derived from Fisher’s exact tests. TN—Treatment-naïve, TEVF—Treatment-experienced with virologic failure.

To further characterise polymorphisms identified in the Manhattan plot, polymorphism prevalence was compared between TN and TE individuals with VF across the RT connection and RNase H domains (Figure 5). In the RT connection, L422K, L425V and G384K were significantly more frequent in the VF group compared to treatment-naïve individuals. L422K showed the highest enrichment, with a marked increase in frequency among failing individuals. Similarly, L425V and G384K were observed at low frequencies in treatment-naïve sequences but were significantly enriched following ART exposure. In the RNase H domain, Q500Y, L503M and Q507Y demonstrated higher prevalence in treatment-experienced individuals with VF. While L503M was present at appreciable frequency in treatment-naïve individuals, it remained significantly more prevalent in the VF group. In contrast, Q500Y and Q507Y were rare or absent in treatment-naïve individuals but were enriched under ART pressure.

### 3.7. Overall Viral Load Distribution in Post-ART Treatment-Naïve and Treatment-Experienced Individuals with Virologic Failure

**Figure 6:**
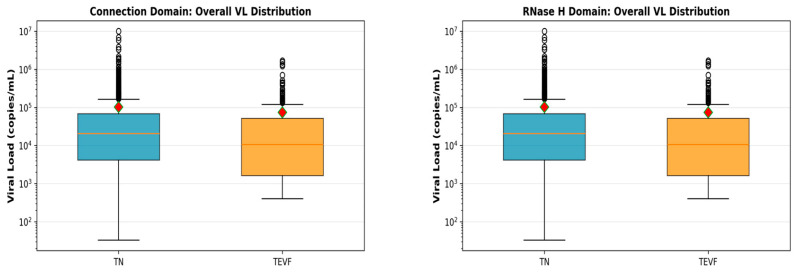
Viral load distributions in post-ART treatment-naïve and treatment-experienced with virologic failure individuals across RT domains: Distribution of viral load values in post-ART treatment-naïve and treatment-experienced with virologic failure across the RT connection and RNase H domains. Viral load is shown on a log_10_ scale. Those with virologic failure demonstrate a marked shift toward higher viral load values compared to treatment-naïve individuals, indicating ongoing viral replication despite ART exposure.

Overall viral load distributions were compared between post-ART TN individuals and TE individuals with VF across sequences spanning the RT connection and RNase H domains (Figure 6). The overall median log_10_ viral load was 4.23 copies/mL (IQR: 3.45–4.80). TN individuals demonstrated a median log_10_ viral load of 4.26 (IQR: 3.53–4.82), whereas TEVF individuals had a median log_10_ viral load of 4.03 (IQR: 3.21–4.71). Comparison using the Wilcoxon rank-sum test yielded a *p*-value of 0.05, indicating a borderline difference between the two groups. Viral load distributions showed substantial overlap.

### 3.8. Regimen-Associated RT Connection and RNase H Polymorphisms

To evaluate regimen-specific selection within the RT connection and RNase H domains, polymorphisms significantly enriched in TE individuals with VF were examined in relation to known NRTI-based treatment regimens (Table 2). All listed polymorphisms showed significantly higher prevalence in the VF group compared to treatment-naïve individuals, with odds ratios ranging from 2.68 to 30.60. Two polymorphisms in the RT connection domain, L422K and G384K, were associated with thymidine analogue-containing regimens, including zidovudine (AZT) and stavudine (d4T). These polymorphisms were rare among treatment-naïve individuals but markedly enriched in treatment-experienced individuals, consistent with strong drug-selective pressure. In contrast, L425V, also located in the connection domain, was primarily associated with tenofovir-containing regimens. Within the RNase H domain, Q500Y, L503M and Q507Y were linked to modern first-line NRTI regimens containing lamivudine or emtricitabine, with or without tenofovir. Q500Y and Q507Y were observed at very low frequencies in treatment-naïve individuals but showed substantial enrichment in the VF group, whereas L503M was present at higher baseline frequency but remained significantly more prevalent in treatment-experienced individuals.

### 3.9. Significant Covariant Analysis

To explore potential cross-domain interactions, covariant analysis was performed across the full RT region, using known polymerase drug resistance mutations (M184V, M184I and D67N) as anchor mutations to identify substitutions that significantly co-occurred within the RT connection and RNase H domains shown in Table 3. All three polymerase mutations exhibited evidence of covariation with substitutions in the RT connection and RNase H domains, with statistical significance assessed using Fisher’s exact test. M184V, although present in a smaller dataset (n = 2357), demonstrated 31 significant pairings. In contrast, M184I (n = 51,869) and D67N (n = 12,189), both more prevalent, exhibited extensive covariant networks with 1294 and 817 significant connections, respectively.

### 3.10. Network Analysis of M184V Reveals Interactions with RT Connection and RNase H Variants

**Figure 7:**
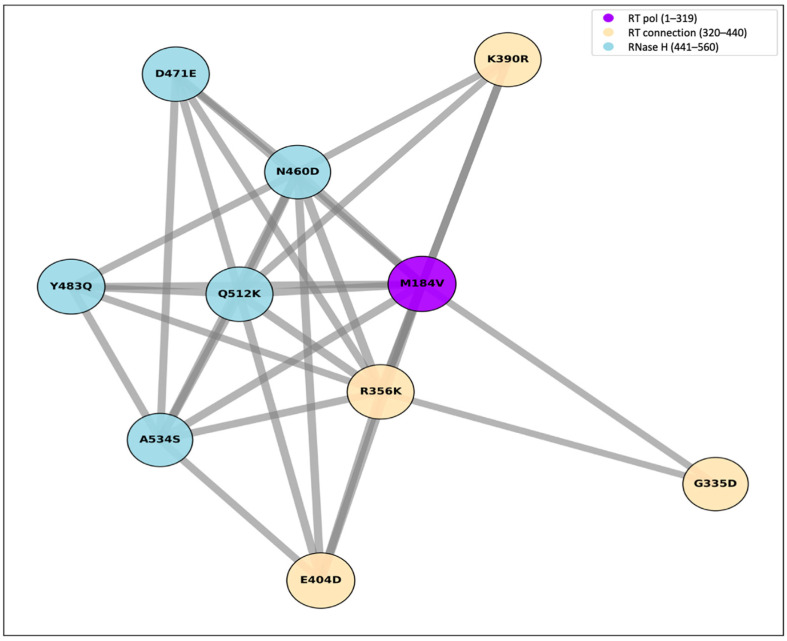
Covariant network of M184V with RT connection and RNase H substitutions: Network representation of significant covariant pairings associated with the M184V mutation (purple node) in the reverse transcriptase polymerase domain. Nodes represent amino acid substitutions located within the polymerase (purple), RT connection (yellow), and RNase H (blue) domains, with connections indicating significant covariation based on Fisher’s exact test. Line thickness reflects the strength of the statistical association.

To further explore cross-domain associations, we focused on M184V, a well-characterised polymerase resistance mutation that yielded a distinct and interpretable network (Figure 7). Despite being represented in a comparatively smaller dataset (n = 2357), M184V demonstrated 31 statistically significant covariant pairings. The resulting network revealed interactions with substitutions distributed across both the RT connection and RNase H domains, including K390R, R356K, E404D, N460D, D471E, Y483Q, Q512K and A534S. The network structure was relatively compact compared with those generated for M184I and D67N yet displayed clear cross-domain connectivity. These findings suggest that M184V, while less frequent, engages in specific covariation patterns that may reflect compensatory or structural interactions beyond the polymerase region.

## 4. Discussion

Our study provides the first comprehensive characterisation of the polymorphism landscape within the HIV-1C RT-conn and RNase H domains in HIV-1C sequences from Botswana. The findings reveal notable variation in naturally occurring polymorphisms across temporal and treatment groups, reflecting both natural viral diversity and the selective pressures exerted by long-term ART exposure.

The presence of certain polymorphisms across eras, such as L491P, M357R, V435I and E449D, suggest that these residues are stably maintained within the circulating HIV-1C population, potentially representing subtype-specific structural or functional adaptations of the RT enzyme [10,17,22,23,24]. The increase in polymorphisms among post-ART TN individuals compared to those from the pre-ART era is suggestive of changes in the natural polymorphism profile of circulating strains; this shift may reflect indirect drug pressure at the population level and/or onward transmission of ART-adapted variants from treated individuals, as has been observed following ART scale-up in resource-limited settings [3,25]. Similar trends have been observed in other sub-Saharan African (SSA) settings; a multi-country study conducted across 11 regions in Kenya, Nigeria, South Africa, Uganda, Zambia, and Zimbabwe among TN populations reported increasing population-level drug resistance with each additional year following ART rollout [26].

Several polymorphisms, such as R461K, L491P and E396D, were present at high frequencies (>10%) before ART introduction, implying that they may be natural polymorphisms rather than drug-associated polymorphisms. However, their continued occurrence post-ART rollout and increase among TE individuals suggest they may influence drug susceptibility or viral enzyme efficacy in the context of HIV-1C [17,27]. Previous studies have shown that RT-conn and RNase H domains can indirectly enhance resistance to RTIs by modulating enzyme processivity, polymerase–RNase H balance or template switching efficiency [4,12,13,14,28,29].

Using a genome-wide association framework across the RT region, specific RT-conn and RNase H polymorphisms were identified that were significantly higher in TE with VF individuals compared to TN individuals. We show that clustering of statistically significant associations within defined RT subdomains indicates non-random patterns of variation. Polymorphisms such as L422K, G384K and L425V in the connection domain, together with Q500Y, L503M and Q507Y in the RNase H domain, exceeded the Bonferroni-corrected threshold and were consistently enriched among individuals TE with VF. These findings imply that specific residues outside the polymerase active site may contribute to sustained viral replication in the presence of ART, potentially through compensatory or modulatory effects on enzyme function [4,28].

We observed a higher prevalence of RT-conn and RNase H polymorphisms in TE with VF than in TN individuals. While some substitutions, such as L503M, were detectable at baseline, others, including Q500Y and Q507Y, were rare or absent among TN individuals and emerged predominantly in those with VF. This pattern supports a model in which certain RT-conn and RNase H polymorphisms are preferentially selected under ART pressure rather than representing background subtype-specific variation [30,31].

Analysis of viral load distributions further supports the observed polymorphism patterns, with TE with VF individuals showing higher viral load values compared to post-ART TN individuals, consistent with ongoing viral replication despite ART exposure [32,33]. This difference in viral load distributions indicate that the enrichment of RT-conn and RNase H polymorphisms occurs in the setting of sustained replication, where selective pressures can actively shape viral genetic composition [34]. The coexistence of elevated viral load and domain-specific polymorphisms supports the hypothesis that these substitutions may facilitate viral persistence under ART pressure through indirect effects on RT activity [35].

Linking polymorphism enrichment to treatment history, regimen-associated analyses demonstrated that distinct RT-conn and RNase H substitutions were observed in individuals exposed to specific NRTI-based regimens. Polymorphisms L422K and G384K were associated with historical thymidine analogue use, whereas L425V was linked to tenofovir-containing regimens. In the RNase H domain Q500Y, L503M and Q507Y were enriched in individuals receiving lamivudine- or emtricitabine-based combinations. These associations suggest that ART regimens exert differential selective pressures across extended domains, resulting in polymorphic signatures that reflect treatment history rather than variation [23,30,36].

Beyond polymorphism effects, covariant analysis showed extensive cross-domain interactions between well-characterised polymerase mutations and distal RT-conn and RNase H variants. Co-occurrence of the RT-conn and RNase H polymorphisms with the classical DRMs such as M184V and D47N imply coordinated evolutionary pathways across RT domains that may sustain viral replication despite drug pressure [17,27,28]. The extent of covariant connections observed for M184I and D67N likely reflects their higher prevalence, whereas the more compact but interpretable M184V network highlights specific, potentially compensatory interactions.

Focusing on M184V, the covariant network revealed statistically significant associations with substitutions distributed across both the RT-conn and RNase H domains, including K390R, R356K, E404D, Q512K and A534S. These cross-domain linkages reinforce the concept of RT as a structurally integrated enzyme, in which alterations in one region may necessitate compensatory adjustments elsewhere to preserve replication capacity under drug pressure [5,37,38]. Such interactions may be particularly relevant in HIV-1C, where baseline polymorphism patterns differ from HIV-1B and could influence evolutionary trajectories [9]. The high frequencies of certain RNase H polymorphisms among failing individuals also identify candidate sites for phenotypic evaluation, which could clarify their contribution to resistance mechanisms and treatment efficacy in HIV-1C [5,17].

These findings are particularly relevant to Botswana, where reverse transcriptase inhibitors (RTIs) remain central to first- and second-line ART regimens. They indicate that genotyping only the polymerase may miss important ART-selected polymorphisms in the RT-conn and RNase H domains, highlighting potential contributors to treatment failure in current ART programmes.

A limitation of this study is the absence of structural data to directly evaluate the effects of the polymorphisms identified in the RT connection and RNase H domains. As the RT polymerase domain is the primary target of reverse transcriptase inhibitors, structural modelling of these regions would not reveal direct effects on drug binding. However, the RT connection and RNase H domains play important roles in reverse transcriptase function, and variations within them may exert indirect effects on viral replication dynamics or antiretroviral susceptibility. Investigating these potential indirect effects through detailed structural and functional studies represents an important avenue for future research.

## 5. Conclusions

The HIV-1C RT-conn and RNase H domains in Botswana show naturally occurring polymorphisms that shift in frequency in the pre-ART and post-ART eras and in TE individuals with VF. The findings demonstrate that these extended RT domains harbour naturally occurring polymorphisms and undergo measurable shifts following widespread ART rollout, reflecting both natural viral diversity and long-term ART pressure. The persistence of several polymorphisms across eras, together with the enrichment of specific RT-conn and RNase H variants in individuals with VF, indicates non-random selection shaped by treatment exposure. Regimen-associated patterns suggest that extended RT domains retain molecular signatures of both historical thymidine analogue-based regimens and contemporary NRTI-containing combinations. These associations were observed in the setting of elevated viral load, supporting their relevance during active viral replication. Covariant analysis further revealed structured covariation between canonical polymerase domain resistance mutations and polymorphisms in the RT-conn and RNase H domains, underscoring the integrated nature of RT evolution under drug pressure. While functional implications cannot be inferred from sequence data alone, these findings identify candidate residue for further phenotypic and structural investigation.

This study supports the expansion of extended RT regions in molecular surveillance frameworks, especially in HIV-1C-dominant settings where NRTI-based regimens remain central to ART. Future work should prioritise phenotypic validation and longitudinal analyses under contemporary treatment regimens to refine subtype-specific resistance interpretation.

## Figures and Tables

**Table 1:** Baseline demographics for included BCPP participants.

Characteristics	Total n = 1495 (%)	Treatment-Naïve n = 1282 (%)	Treatment-Experienced with Virologic Failure n = 213 (%)	*p*-Value
District				0.01
Northern	269 (18.0%)	216 (16.8%)	53 (24.9%)	
Southern	574 (38.4%)	504 (39.3%)	70 (32.9%)	
Central	652 (43.6%)	562 (43.8%)	90 (42.2%)	
Gender				0.6
Male	501 (29.2%)	426 (33.2%)	75 (35.2%)	
Female	994 (70.8%)	856 (66.8%)	138 (64.8%)	
Median age in years				
(Q_1_, Q_3_)	34.0 (27.0, 42.0)	34.0 (27.2, 42.0)	34 (26.7, 41.0)	0.6 **
Median log_10_ (viral load)				
(Q_1_, Q_3_)	4.23 (3.45, 4.80)	4.26 (3.53, 4.82)	4.03 (3.21, 4.71)	0.05 **

n = Sample size, Q1—First quartile, Q3—Third quartile, ** *p*-values obtained using Wilcoxon rank-sum test while other *p*-values were from Chi-square test. Numbers in brackets are percentages within columns.

**Table 2:** Regimen-associated RT connection and RNase H polymorphisms enriched in treatment-experienced individuals with virologic failure.

Polymorphism	Domain	Key Selecting Regimen(s)	Drug Class	TAM-Associated	TN vs. TEVF Odds Ratio	Fitness Cost	Clinical Interpretation
L422K	Connection	AZT, d4T	NRTI (TAMs)	Yes	6.05	Moderate	Marker of historical thymidine analogue exposure
G384K	Connection	AZT, d4T	NRTI (TAMs)	Yes	10.86	High	Rare TAM-related polymorphism indicating older regimens
L425V	Connection	TDF	NRTI	No	5.73	Moderate	Associated with tenofovir-containing regimens
Q500Y	RNase H	3TC, FTC	NRTI	No	20.87	High	Strong marker of modern ART failure
L503M	RNase H	3TC, FTC, TDF	NRTI	No	2.68	Low	Naturally occurring polymorphism under ART selection
Q507Y	RNase H	3TC, FTC	NRTI	No	30.6	Very High	Highly ART-specific polymorphism, absent in untreated

AZT—zidovudine, d4T—ztavudine, TDF—tenofovir disoproxil fumarate, 3TC—lamivudine, FTC—emtricitabine, NRTI—nucleoside reverse transcriptase inhibitor, TAMs—thymidine analogue mutations.

**Table 3:** Significant covariant analysis of known RT polymerase mutations with RT connection and RNase H substitutions.

Name of Mutation	Total Rows (Samples) Processed	Covariant (Significant Pairs) Found	Number of Significant Connections After Fisher’s Tests
M184V	2357	Yes	31
M184I	51,869	Yes	1294
D67N	12,189	Yes	817

## Data Availability

The data presented in this study are not publicly available due to ethical restrictions. De-identified data may be available from the corresponding author upon reasonable request and with approval from the relevant institutional review boards.

## Abbreviations

The following abbreviations are used in this manuscript:

HIV	Human immunodeficiency virus
SSA	Sub-Saharan Africa
HIV-1C	Human immunodeficiency virus type 1 subtype C
ART	Antiretroviral therapy
LMICs	Low- and middle-income countries
PWH	People with HIV
HIV-1B	Human immunodeficiency virus type 1 subtype B
RT	Reverse transcriptase
NRTIs	Nucleoside reverse transcriptase inhibitors
NNRTIs	Non-nucleoside reverse transcriptase inhibitors
NRTTIs	Nucleoside reverse transcriptase translocation inhibitors
RTIs	Reverse transcriptase inhibitors
RT-conn	Reverse transcriptase connection
RNase H	Ribonuclease H
DRMs	Drug resistance mutations
NVP	Nevirapine
DLV	Delavirdine
TN	Treatment-naïve
TE	Treatment-experienced
VF	Virologic failure
BCPP	Botswana Combination Prevention Project
EFV	Efavirenz
DTG	Dolutegravir
NGS	Next-generation sequencing
RNA	Ribonucleic acid
DNA	Deoxyribonucleic acid
VL	Viral load
VS	Virally suppressed
MSA	Multiple-sequence alignment
IQRs	Interquartile ranges

## References

[1] Cohen M.S., Chen Y.Q., McCauley M., Gamble T., Hosseinipour M.C., Kumarasamy N., Hakim J.G., Kumwenda J., Grinsztejn B., Pilotto J.H. (2011). Prevention of HIV-1 infection with early antiretroviral therapy. N. Engl. J. Med..

[2] Stoneburner R., Korenromp E., Lazenby M., Tassie J.M., Letebele J., Motlapele D., Granich R., Boerma T., Low-Beer D. (2014). Using health surveillance systems data to assess the impact of AIDS and antiretroviral treatment on adult morbidity and mortality in Botswana. PLoS ONE.

[3] Gupta R.K., Jordan M.R., Sultan B.J., Hill A., Davis D.H., Gregson J., Sawyer A.W., Hamers R.L., Ndembi N., Pillay D. (2012). Global trends in antiretroviral resistance in treatment-naive individuals with HIV after rollout of antiretroviral treatment in resource-limited settings: A global collaborative study and meta-regression analysis. Lancet.

[4] Hachiya A., Shimane K., Sarafianos S.G., Kodama E.N., Sakagami Y., Negishi F., Koizumi H., Gatanaga H., Matsuoka M., Takiguchi M. (2009). Clinical relevance of substitutions in the connection subdomain and RNase H domain of HIV-1 reverse transcriptase from a cohort of antiretroviral treatment-naïve patients. Antivir. Res..

[5] von Wyl V., Ehteshami M., Demeter L.M., Bürgisser P., Nijhuis M., Symons J., Yerly S., Böni J., Klimkait T., Schuurman R. (2010). HIV-1 reverse transcriptase connection domain mutations: Dynamics of emergence and implications for success of combination antiretroviral therapy. Clin. Infect. Dis..

[6] Dau B., Ayers D., Singer J., Harrigan P.R., Brown S., Kyriakides T., Cameron D.W., Angus B., Holodniy M. (2010). Connection domain mutations in treatment-experienced patients in the OPTIMA trial. J. Acquir. Immune Defic. Syndr..

[7] Soares E.A., Makamche M.F., Siqueira J.D., Lumngwena E., Mbuagbaw J., Kaptue L., Asonganyi T., Seuánez H.N., Soares M.A., Alemnji G. (2010). Molecular diversity and polymerase gene genotypes of HIV-1 among treatment-naïve Cameroonian subjects with advanced disease. J. Clin. Virol..

[8] Santos A.F., Silveira J., Muniz C.P., Tornatore M., Góes L.R., Mendoza-Sassi R.A., Martinez A.M., Tupinambás U., Greco D.B., Soares M.A. (2011). Primary HIV-1 drug resistance in the C-terminal domains of viral reverse transcriptase among drug-naïve patients from Southern Brazil. J. Clin. Virol..

[9] Martinez-Cajas J.L., Pai N.P., Klein M.B., Wainberg M.A. (2009). Differences in resistance mutations among HIV-1 non-subtype B infections: A systematic review of evidence (1996–2008). J. Int. AIDS Soc..

[10] Wensing A.M., Calvez V., Ceccherini-Silberstein F., Charpentier C., Günthard H.F., Paredes R., Shafer R.W., Richman D.D. (2022). 2022 update of the drug resistance mutations in HIV-1. Top Antivir. Med..

[11] Cilento M.E., Wen X., Reeve A.B., Ukah O.B., Snyder A.A., Carrillo C.M., Smith C.P., Edwards K., Wahoski C.C., Kitzler D.R. (2023). HIV-1 Resistance to Islatravir/Tenofovir Combination Therapy in Wild-Type or NRTI-Resistant Strains of Diverse HIV-1 Subtypes. Viruses.

[12] Delviks-Frankenberry K.A., Nikolenko G.N., Barr R., Pathak V.K. (2007). Mutations in human immunodeficiency virus type 1 RNase H primer grip enhance 3′-azido-3′-deoxythymidine resistance. J. Virol..

[13] Nikolenko G.N., Palmer S., Maldarelli F., Mellors J.W., Coffin J.M., Pathak V.K. (2005). Mechanism for nucleoside analog-mediated abrogation of HIV-1 replication: Balance between RNase H activity and nucleotide excision. Proc. Natl. Acad. Sci. USA.

[14] Nikolenko G.N., Delviks-Frankenberry K.A., Pathak V.K. (2010). A novel molecular mechanism of dual resistance to nucleoside and nonnucleoside reverse transcriptase inhibitors. J. Virol..

[15] Munerato P., Sucupira M.C., Oliveros M.P., Janini L.M., de Souza D.F., Pereira A.A., Inocencio L.A., Diaz R.S. (2010). HIV type 1 antiretroviral resistance mutations in subtypes B, C, and F in the City of São Paulo, Brazil. AIDS Res. Hum. Retroviruses.

[16] Muniz C.P., Soares M.A., Santos A.F. (2014). Early selection of resistance-associated mutations in HIV-1 RT C-terminal domains across different subtypes: Role of the genetic barrier to resistance. J. Antimicrob. Chemother..

[17] Barral M.F.M., Sousa A.K.P., Santos A.F., Abreu C.M., Tanuri A., Soares M.A. (2017). Identification of Novel Resistance-Related Polymorphisms in HIV-1 Subtype C RT Connection and RNase H Domains from Patients Under Virological Failure in Brazil. AIDS Res. Hum. Retroviruses.

[18] Elangovan R., Jenks M., Yun J., Dickson-Tetteh L., Kirtley S., Hemelaar J. (2021). Global and Regional Estimates for Subtype-Specific Therapeutic and Prophylactic HIV-1 Vaccines: A Modeling Study. Front Microbiol..

[19] Hadfield J., Megill C., Bell S.M., Huddleston J., Potter B., Callender C., Sagulenko P., Bedford T., Neher R.A. (2018). Nextstrain: Real-time tracking of pathogen evolution. Bioinformatics.

[20] Novitsky V., Zahralban-Steele M., McLane M.F., Moyo S., van Widenfelt E., Gaseitsiwe S., Makhema J., Essex M. (2015). Long-Range HIV Genotyping Using Viral RNA and Proviral DNA for Analysis of HIV Drug Resistance and HIV Clustering. J. Clin. Microbiol..

[21] Anderson M., Gaseitsiwe S., Moyo S., Thami K.P., Mohammed T., Setlhare D., Sebunya T.K., Powell E.A., Makhema J., Blackard J.T. (2016). Slow CD4^+^ T-Cell Recovery in Human Immunodeficiency Virus/Hepatitis B Virus-Coinfected Patients Initiating Truvada-Based Combination Antiretroviral Therapy in Botswana. Open Forum Infect. Dis..

[22] Novitsky V., Zahralban-Steele M., Moyo S., Nkhisang T., Maruapula D., McLane M.F., Leidner J., Bennett K., Abeler-Dörner L., E Wirth K. (2020). Mapping of HIV-1C Transmission Networks Reveals Extensive Spread of Viral Lineages Across Villages in Botswana Treatment-as-Prevention Trial. J. Infect. Dis..

[23] Santos A.F., Lengruber R.B., Soares E.A., Jere A., Sprinz E., Martinez A.M.B., Silveira J., Sion F.S., Pathak V.K., Soares M.A. (2008). Conservation patterns of HIV-1 RT connection and RNase H domains: Identification of new mutations in NRTI-treated patients. PLoS ONE.

[24] Wainberg M.A., Brenner B.G. (2012). The Impact of HIV Genetic Polymorphisms and Subtype Differences on the Occurrence of Resistance to Antiretroviral Drugs. Mol. Biol. Int..

[25] Rhee S.Y., Kassaye S.G., Barrow G., Sundaramurthi J.C., Jordan M.R., Shafer R.W. (2020). HIV-1 transmitted drug resistance surveillance: Shifting trends in study design and prevalence estimates. J. Int. AIDS Soc..

[26] Hamers R.L., Wallis C.L., Kityo C., Siwale M., Mandaliya K., Conradie F., Botes E.M., Wellington M., Osibogun A., Sigaloff E.K.C. (2011). HIV-1 drug resistance in antiretroviral-naive individuals in sub-Saharan Africa after rollout of antiretroviral therapy: A multicentre observational study. Lancet Infect. Dis..

[27] Delviks-Frankenberry K.A., Lengruber R.B., Santos A.F., Silveira J.M., Soares M.A., Kearney M.F., Maldarelli F., Pathak V.K. (2013). Connection subdomain mutations in HIV-1 subtype-C treatment-experienced patients enhance NRTI and NNRTI drug resistance. Virology.

[28] Brehm J.H., Koontz D., Meteer J.D., Pathak V., Sluis-Cremer N., Mellors J.W. (2007). Selection of mutations in the connection and RNase H domains of human immunodeficiency virus type 1 reverse transcriptase that increase resistance to 3′-azido-3′-dideoxythymidine. J. Virol..

[29] Beilhartz G.L., Götte M. (2010). HIV-1 Ribonuclease H: Structure, Catalytic Mechanism and Inhibitors. Viruses.

[30] Delviks-Frankenberry K.A., Nikolenko G.N., Pathak V.K. (2010). The “Connection” Between HIV Drug Resistance and RNase H. Viruses.

[31] Ngcapu S., Theys K., Libin P., Marconi V.C., Sunpath H., Ndung’u T., Gordon M.L. (2017). Characterization of Nucleoside Reverse Transcriptase Inhibitor-Associated Mutations in the RNase H Region of HIV-1 Subtype C Infected Individuals. Viruses.

[32] Martinez-Picado J., Deeks S.G. (2016). Persistent HIV-1 replication during antiretroviral therapy. Curr. Opin. HIV AIDS.

[33] Pennings P.S. (2012). Standing genetic variation and the evolution of drug resistance in HIV. PLoS Comput. Biol..

[34] Zhou S., Long N., Swanstrom R. (2023). Evolution Driven By A Varying Host Environment Selects For Distinct HIV-1 Entry Phenotypes and Other Informative Variants. Front. Virol..

[35] van Zyl G., Bale M.J., Kearney M.F. (2018). HIV evolution and diversity in ART-treated patients. Retrovirology.

[36] Wright D.W., Deuzing I.P., Flandre P., van den Eede P., Govaert M., Setiawan L., Coveney P.V., Marcelin A.-G., Calvez V., Boucher C.A.B. (2013). A polymorphism at position 400 in the connection subdomain of HIV-1 reverse transcriptase affects sensitivity to NNRTIs and RNaseH activity. PLoS ONE.

[37] Theys K., Deforche K., Libin P., Camacho R.J., Van Laethem K., Vandamme A.M. (2010). Resistance pathways of human immunodeficiency virus type 1 against the combination of zidovudine and lamivudine. J. Gen. Virol..

[38] Biswas A., Haldane A., Arnold E., Levy R.M. (2019). Epistasis and entrenchment of drug resistance in HIV-1 subtype B. Elife.

